# Does physical exercise inhibit smoking behavior? Evidence from the China General Social Survey

**DOI:** 10.3389/fpubh.2025.1723830

**Published:** 2026-01-06

**Authors:** Shutong Zhao, Haiyan Huang

**Affiliations:** 1 College of Physical Education, West Anhui University, Lu’an, China; 2 School of Economics and Management, Shanghai University of Sport, Shanghai, China; 3 School of Management, Beijing Sport University, Beijing, China

**Keywords:** China General Social Survey, mental health, physical exercise, psychological effects, smoking behavior, social interaction, social support

## Abstract

**Introduction:**

Examining how physical exercise influences smoking behavior and the mediating mechanisms involved helps clarify the broader social benefits of exercise and informs efforts to improve population health and build a more harmonious society.

**Methods:**

This cross-sectional observational study analyzed the nationally representative 2021 China General Social Survey (CGSS). After sample screening, we retained 2,373 valid observations. We estimated OLS models to examine the association between physical exercise and residents’ smoking behavior. For robustness, we re-estimated ordered probit and ordered logit specifications and used propensity score matching (PSM) as an endogeneity check against the baseline estimates. We further assessed mediation by mental health and social support and tested the moderating role of individual income.

**Results:**

The results showed a significant positive association between physical exercise and reduced smoking (*β* = 0.0276), which remained robust after sensitivity and endogeneity checks. Subgroup analyses indicated heterogeneity: the association was significant at the 1% level for middle-aged adults and at the 10% level for younger adults, but not for older adults. For both men and women, the association was statistically significant at the 5% level, with a larger coefficient for men than for women (0.0359 and 0.0167). Mediation tests suggested that improvements in mental health and increases in social support were key pathways through which exercise was associated with lower smoking. Moderation analyses further showed that income positively moderated the exercise–smoking association, with higher income strengthening this association.

**Conclusion:**

Physical exercise is associated with reduced smoking, and the mechanisms of mental health and social support help explain this association. These findings deepened understanding of how exercise relates to smoking behavior and provided practical implications for public health policy and tobacco-control interventions in China. The study also extended research on the social and psychological effects of exercise and offered a theoretical basis for future work. However, because the analysis is cross-sectional and relies on self-reported measures from a single country and a single-year dataset, the findings are correlational and context-specific.

## 1 Introduction

Tobacco harm is among the most serious global public health challenges (1). Tobacco use causes approximately 8.7 million deaths annually (2). Tobacco dependence is implicated in the onset and progression of diabetes, stroke, coronary heart disease, malignant neoplasms, and chronic obstructive pulmonary disease (3). In 2019, the global number of smokers reached an estimated 1.14 billion, the majority residing in low- and middle-income countries (4). Prior research also indicates that smoking is more prevalent among disadvantaged populations, such as those with lower educational attainment (5). Moreover, physical exercise, an important behavior for improving physical health and psychological well-being, has been regarded as an effective means of intervening in unhealthy habits (6). In recent years, growing attention has been paid to whether physical exercise can, to some extent, suppress smoking behavior, particularly within frameworks of public health interventions and behavioral substitution mechanisms (7). International studies suggest a potential negative association between physical exercise and smoking behavior. On the one hand, individuals who engage in exercise typically adopt healthier lifestyles and are more likely to avoid or quit smoking. On the other hand, physical exercise can alleviate stress and improve mood, thereby diminishing dependence on smoking. For example, a study of healthcare workers in Pakistan found that higher stress levels were linked to greater smoking risk, whereas engaging in exercise significantly attenuated this association (8). Moreover, exercise may delay the initiation or escalation of smoking through psychophysiological pathways. Among adolescents, greater weekly exercise frequency has been associated with a reduced likelihood of smoking and lower smoking intensity among current smokers (9). Experimental evidence further indicates that high-intensity exercise can increase *β*-endorphin levels and postpone the onset of cigarette craving, suggesting a potential adjunctive role for exercise in smoking cessation (10). Nevertheless, important limitations remain in the literature: evidence for Chinese adults is relatively scarce and often based on non-representative or localized samples; potential mechanisms are seldom examined jointly within the same empirical framework; and socioeconomic stratification, particularly whether income conditions the exercise-smoking association, has received limited attention. Furthermore, many studies rely on a single estimator with few checks for confounding, leaving concerns about robustness and identification.

Although prior research suggests that physical exercise is often negatively associated with smoking behavior, these associations may vary across countries, cultural contexts, and social environments. In China, amid rapid social transformation and shifting health behaviors, evidence on the correlational patterns between exercise and smoking among adults remains limited, and the putative pathways warrant further examination. To align with the observational, cross-sectional nature of CGSS 2021, our objective is to describe the association between exercise and smoking and to explore whether mental health and social support statistically account for part of this association, as well as whether it varies by income, age, and gender. Clarifying these gaps is theoretically and practically salient: doing so can specify the positive social externalities of sport participation, indicate for whom exercise matters most, and inform targeted cessation policies under Healthy China 2030. To address these limitations, this study uses nationally representative data from CGSS 2021; triangulates ordinary least squares with ordered probit/logit and propensity score matching to enhance robustness and mitigate selection on observables; tests two mediating pathways (mental health and social support) within a single framework; and examines heterogeneity by age and gender as well as the moderating role of individual income. By jointly identifying direct, indirect, and moderated associations in a large adult Chinese sample, we contribute new evidence on mechanisms and socioeconomic contingencies that have been underexplored in prior work, while offering policy-relevant effect estimates for smoking-cessation strategies in China.

This study made three main contributions. First, it offered a detailed, multi-faceted examination of the relationship between physical exercise and smoking behavior. Using OLS, we introduced individual, household, and regional covariates stepwise and explicitly accounted for heterogeneity across populations by examining the effect of physical exercise on smoking behavior across gender and age groups. This study provides a more comprehensive and nuanced understanding.

Second, to ensure the robustness of the findings, we employed multiple econometric strategies to address potential endogeneity and self-selection bias. By varying model specifications, re-operationalizing the key explanatory variable, adding additional controls, and applying PSM, we mitigated concerns about reverse causality and nonrandom participation. These rigorous approaches strengthened the credibility and external validity of the results.

Third, to clarify the mediating and moderating mechanisms through which physical exercise affects smoking behavior, we estimated mediation and moderation models. Treating mental health and social support as potential mediators, we identified indirect channels associated with exercise and smoking behavior. This study further examined whether individual income moderates the association between physical exercise and smoking behavior. The evidence offered practical insights for designing targeted public health policies and smoking-cessation interventions in China.

### 1.1 Relationship between physical exercise and smoking behavior

A substantial body of evidence indicates that regular physical exercise is associated with lower smoking behavior. An observational study of more than 29,000 individuals found that those who exercise have a markedly lower smoking prevalence, and higher exercise frequency is associated with a reduced probability of smoking, with the effect especially pronounced among men (11). Epidemiological observational studies likewise suggest lower odds of smoking among regular exercisers and higher cravings among the physically inactive (12). In a longitudinal study of 1,374 adolescents, Audrain-McGovern et al. (13) reported that one pathway through which physical activity is associated with lower uptake or intensity of smoking involves reward-related mechanisms, and this longitudinal design offers stronger causal leverage than cross-sectional surveys. Mechanistically, physical activity may partly substitute for the hedonic effects of nicotine and attenuate discomfort during cessation. Researchers have observed that experimental and clinical studies find that exercise can function as a nicotine substitute, often increasing quit intentions, alleviating withdrawal symptoms, and reducing short-term craving (11). However, high-quality evidence also reports null or uncertain effects on long-term abstinence: a 2019 Cochrane Review found no evidence that adding exercise to standard cessation support improves sustained abstinence. A meta-analysis of randomized controlled trials reported no effect of aerobic or resistance exercise on cessation; a pragmatic RCT in the UK (TARS) observed no long-term effects on smoking cessation. A recent systematic review concluded that long-term exercise interventions do not significantly improve cessation rates, despite short-term relief of cravings and withdrawal (14–16). Thus, trials and longitudinal designs provide stronger inference.

Accordingly, physical exercise is regarded as a promising intervention that may, in some settings, influence smoking behavior by improving physiological and psychological states, whereas observational studies like ours are framed as associational evidence. Accordingly, we propose Hypothesis H1: Physical exercise is positively associated with reduced smoking behavior.

### 1.2 Mediating mechanisms linking physical exercise to smoking behavior

If H1 is supported, what mechanisms account for the effect of physical exercise on smoking behavior? Only by developing a comprehensive understanding of the functions of exercise can we identify appropriate mediators and, in turn, clarify the pathways through which exercise influences smoking behavior.

#### 1.2.1 Enhancing mental health

Mental health plays a mediating role in smoking behavior. Negative affect and psychological stress are often associated with an increased propensity to smoke as a means of mood regulation, which helps explain the observed linkage between smoking and mental health (17). Empirical evidence shows that depressive and anxious symptomatology is positively associated with higher smoking prevalence and intensity, and individuals with low mood are more likely to initiate smoking or increase consumption (18). Physical exercise, treated here as a correlational exposure rather than a causal intervention, is associated with better mental health (19). Regular activity is linked to the release of neurotransmitters such as endorphins, improved mood, higher self-esteem, and lower stress and depressive symptoms (20). Studies indicate that people who consistently engage in physical activity report more positive affect, lower daily stress, and reduced depressive scores (21). Among smokers, these psychological benefits may lessen the need to smoke to modulate emotions. A large cross-sectional study of healthcare workers in Pakistan further found that high-stress environments were associated with greater smoking risk, whereas regular exercise significantly reduced the odds of smoking; exercise also mediated the link between perceived stress and smoking, and individuals exercising three or more times per week had a markedly lower probability of smoking (8). In short, exercise may provide a healthy coping mechanism that can substitute for the affective relief provided by cigarettes, help stabilize mood, and thereby weaken the mental-health–driven urge to smoke (22). Consequently, exercise may be indirectly associated with lower smoking via improvements in mental health.

Accordingly, we propose Hypothesis H2: Physical exercise is associated with smoking behavior through the mediation of mental health.

#### 1.2.2 Strengthening social support

Social support is widely discussed as an important psychosocial correlate of smoking behavior (23). Social network theory posits that the complex ties among individuals within a given population shape members’ behaviors (24). Smoking appears to be subject to network influences: the degree of support from family, friends, and the community is associated with confidence in quitting and the likelihood of success (25). Participation in cessation support groups and encouragement from close others have been associated with improved short-term abstinence in some studies (23). For example, among 928 smokers enrolled in group-based cessation programs, those who reported “having someone to rely on” were more likely to remain abstinent at 4 weeks (26); couples or intimate partners quitting together reported higher short-term success rates (58%) than individuals attempting to quit alone (38%) (27). These patterns are consistent with the idea that networks provide emotional support and transmit group norms that shape attitudes toward smoking; individuals embedded in cessation circles may be more likely to quit successfully, whereas those surrounded by smoking peers tend to maintain the habit. Physical exercise often has a social dimension, as many activities are undertaken in teams or groups. Such contexts may create opportunities to build positive social ties and receive support (28). Individuals who join sports clubs or engage in group exercise often report forming like-minded friendships and receiving emotional and behavioral support, as well as accountability (29). Some interventions suggest that when exercise is accompanied by social encouragement from workout partners (e.g., teammates), smokers may be better able to counter cravings and strengthen their commitment to “not smoke from today” (30). The social support networks that may grow out of exercise may help alleviate stress, potentially substitute for some socially cued smoking situations, and help reinforce self-control through others’ oversight (31). The social-support enhancement associated with exercise may constitute a mediating pathway linking physical activity to reduced smoking.

Accordingly, we propose Hypothesis H3: Physical exercise is associated with smoking behavior through the mediation of social support.

### 1.3 Moderating role of individual income

Socioeconomic status, particularly personal income, may moderate both the direction and magnitude of the association between physical exercise and smoking behavior (32). In general, low-income individuals (e.g., those living below the poverty line) exhibit higher smoking prevalence and lower cessation success rates than their higher-income counterparts (33). Evidence from intervention settings suggests that, while combining moderate-intensity exercise with cessation support can be beneficial for some, real-world uptake is often low, especially among low-income groups, thereby constraining population impact (34). Conversely, higher-income individuals tend to report healthier lifestyles, smoke less, and exercise more (35). The association between income and health behaviors reflects multiple mechanisms: those with greater financial resources typically possess more health-related knowledge and access to supportive resources, potentially making it easier to leverage exercise for health improvement (36). Conversely, individuals facing financial strain experience greater life stress and may rely on smoking for stress relief (37), and often lack the time or resources to engage in regular exercise. Empirical findings on income-related differences in lifestyle intervention effects are context-dependent but frequently indicate smaller impacts in low-income populations and comparatively larger impacts among higher-income groups (32, 38). These patterns are consistent with the view that personal income positively moderates the association between exercise participation and reduced smoking; as income increases, the observed anti-smoking association of exercise tends to be stronger.

Accordingly, we propose Hypothesis H4: The association between physical exercise and reduced smoking is positively moderated by individual income.

**Figure 1:**
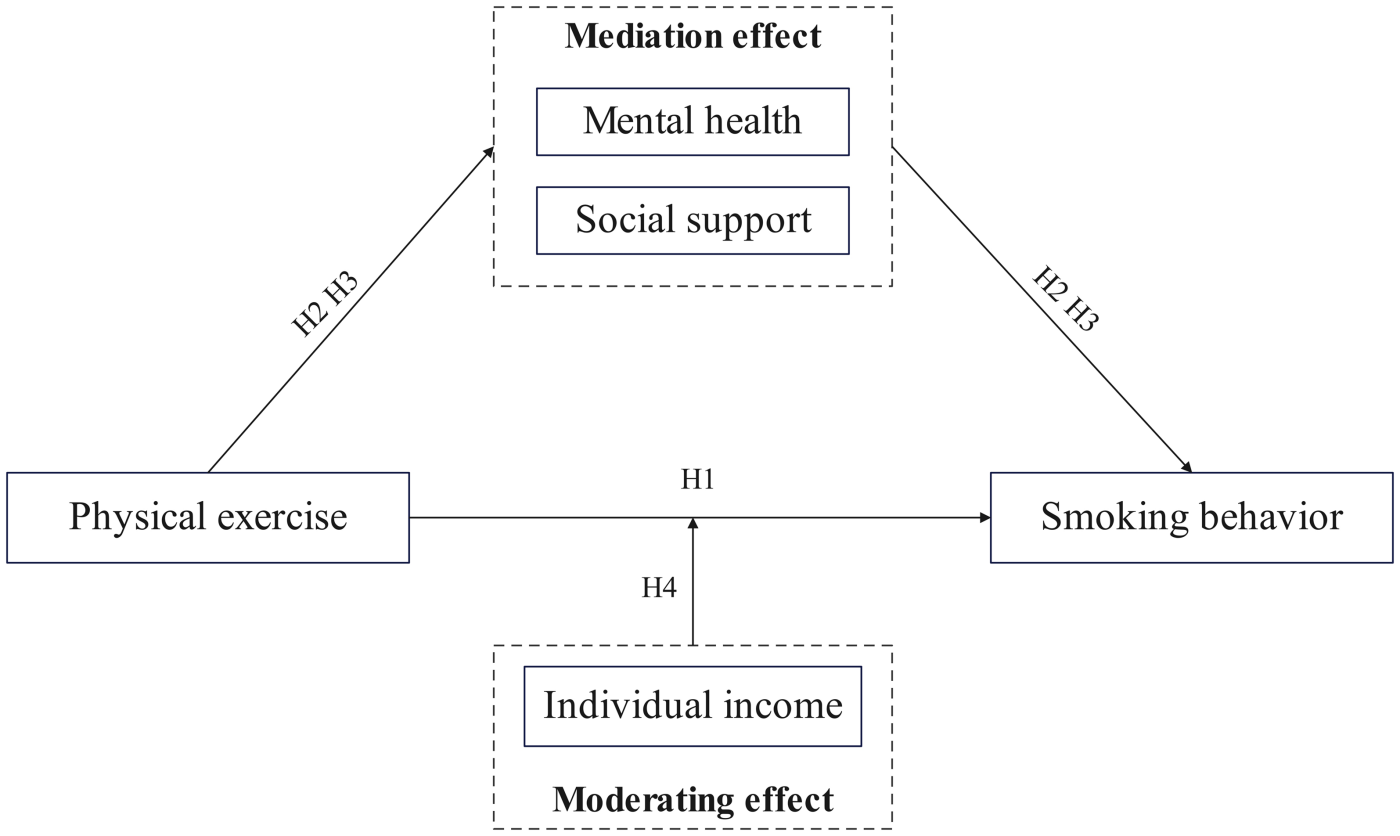
Conceptual model.

Building on the foregoing theoretical analysis and hypotheses, we developed a theoretical framework for the relationship between physical exercise and smoking behavior (Figure 1).

## 2 Research design

### 2.1 Data sources

Data for this study were drawn from the Chinese General Social Survey (CGSS), a nationally representative, cross-sectional survey of individuals in mainland China. Administered by the Department of Sociology at Renmin University of China, the CGSS is publicly available and was launched with its baseline wave in 2003. The survey systematically collected multilevel information at the social, community, family, and individual levels to document patterns of social transformation. Using a multistage, stratified, probability-proportional-to-size (PPS) sampling design, it interviewed approximately 10,000 respondents across 125 counties. Because several key measures for this study (e.g., smoking behavior) were collected only in 2021, we restricted our analysis to the CGSS 2021 wave, which surveyed 8,148 respondents across 31 provinces. To produce an accurate and comprehensive sample profile, we excluded cases with item nonresponse (“Do not know”/“Refused”) and observations with missing values, outliers, or other anomalies in the variables for smoking behavior, physical exercise, mental health, and social support. The final analytic sample comprised 2,373 respondents, offering strong real-world representativeness and analytic utility. All analyses were conducted in Stata 18.0. Descriptive characteristics of the sample are reported in Table 1.

**Table 1:** Demographic characteristics of the sample (*N* = 2,373).

Characteristics	Category	Frequency	Percentage %
Gender	Male	1,091	45.98
	Female	1,282	54.02
Age	Youth (18–34 years old)	464	19.55
	Middle-aged adults (35–64 years old)	1,227	51.71
	Older adults (≥65 years old)	682	28.74
Marriage status	Married	633	26.68
	Unmarried	1,740	73.32
Educational attainment	Primary school or below	802	33.80
	Junior high	667	28.11
	High school	420	17.70
	University or above	484	20.40
Household registration	Rural	1,406	59.25
	Urban	967	40.75
Religious belief	Yes	207	8.72
	No	2,166	91.28
Region of residence	Eastern region	860	36.24
	Central region	778	32.79
	Western region	617	26.00
	Northeast region	118	4.97

### 2.2 Variable selection

#### 2.2.1 Dependent variable

Smoking behavior served as the dependent variable. It was measured using the China General Social Survey (CGSS) item “Do you smoke?,” with response options coded as 1 = currently smoke, 2 = used to smoke but not now, and 3 = never smoked. For empirical analyses, we treated this item as an approximately continuous score, with higher values indicating lower smoking involvement.

#### 2.2.2 Independent variable

The independent variable is physical exercise. We used the CGSS item, “Over the past year, did you regularly engage in physical exercise during your leisure time?” Responses were coded 0–4: 0 = never, 1 = several times per year or less, 2 = several times per month, 3 = several times per week, and 4 = every day. Higher values indicate greater participation in exercise.

#### 2.2.3 Mediator variable

Mental health was specified as the mediating variable. It was measured using the CGSS item, “During the past four weeks, how often have you felt depressed or down?” Responses were recorded on a five-point scale: 1 = always; 2 = often; 3 = sometimes; 4 = seldom; 5 = never. We treated this item as a continuous measure coded 1–5, with higher values indicating better mental health.

Social support was specified as the mediating variable. Drawing on the CGSS, we measured social support with the item, “In the past year, how often did you meet with friends during your leisure time?” Responses were coded on a five-point scale: 1 = “Never,” 2 = “Several times a year or less,” 3 = “Several times a month,” 4 = “Several times a week,” and 5 = “Every day.” Higher scores indicate a greater level of social support.

#### 2.2.4 Moderating variable

Individual income was specified as the moderating variable. Following the CGSS, we used the item: “What was your total personal income for the previous year (2020)?” Responses were recorded as a continuous measure, with higher values indicating higher income.

#### 2.2.5 Control variables

Prior research indicated that sociodemographic and socioeconomic factors also shaped smoking behavior (39). To mitigate omitted-variable bias, we included individual-, household-, and region-level characteristics as control variables in the regression models. Individual characteristics comprised gender, age, age squared, household registration (hukou), religious belief, and educational attainment. Household characteristics included marital status, a household income rating, and household risk investment status. Regional characteristics included GDP per capita and regional per capita disposable income. Detailed variable definitions and codings are reported in Table 2. The descriptive statistics reveal meaningful dispersion across variables, reflecting heterogeneity among Chinese residents at the individual, household, and regional levels, thereby providing an empirical basis for the subsequent analyses.

**Table 2:** Variable definitions and descriptive statistics (*N* = 2,373).

Variable types	Variables	Definition	mean	sd	min	max
Dependent variable	Smoking behavior	1 = Currently smoke, 2 = Used to smoke but not now, and 3 = Never smoke	2.437	0.840	1	3
Independent variable	Physical exercise	0 = Never, 1 = Several times a year or less, 2 = Several times a month, 3 = Several times a week, 4 = Every day	0.771	0.420	0	1
Mediating variables	Mental health	1 = Always, 2 = Often, 3 = Sometimes, 4 = Seldom, 5 = Never	2.885	0.784	1	4
	Social support	1 = Never, 2 = Several times a year or less, 3 = Several times a month, 4 = Several times a week, 5 = Every day	3.925	1.070	1	5
Moderating variable	Individual income	Respondent’s total income in the previous year (2020), 10,000 CNY	2.273	1.050	1	5
Control variables	Gender	1 = Male, 0 = Female	0.460	0.498	0	1
	Age	1 = Youth (18–34 years old), 2 = Middle-aged adults (35–64 years old), 3 = Older adults (≥65 years old)	2.092	0.689	1	3
	Age^2	Age^2/100	30.43	17.98	3.24	98.01
	Household registration	1 = Rural, 0 = Urban	0.592	0.491	0	1
	Religious belief	1 = No, 0 = Yes	0.913	0.282	0	1
	Educational attainment	1 = Primary school or below, 2 = junior high, 3 = high school, 4 = University or above	2.247	1.127	1	4
	Marriage status	1 = Married, 0 = Unmarried and other	0.733	0.442	0	1
	Household income rating	1 = Far below average, 2 = Below average, 3 = Average, 4 = Above average, 5 = Far above average	2.606	0.755	1	5
	Household risk investment status	1 = No, 0 = Yes	0.887	0.317	0	1
	GDP per capita	ln(GDP per capita)	11.29	0.376	10.62	12.14
	Regional per capita disposable income	ln(regional per capita disposable income)	10.46	0.309	10.00	11.23

### 2.3 Model specification

#### 2.3.1 Baseline model

To examine the effect of physical exercise on smoking behavior, we estimated the following baseline regression model:


$$Y_{i}=a_{0}+a_{1}X_{i}+a_{2}C_{i}+\varepsilon_{i}$$ (1)

Where $Y_{i}$ denotes the smoking behavior; $X_{i}$ denotes the participation in physical exercise; $C_{i}$ is the vector of control variables; $a_{0}$ , $a_{1}$ , and $a_{2}$ are parameters to be estimated; and $\varepsilon_{i}$ is the random disturbance term.

#### 2.3.2 Mediation effect model

The effect of physical exercise on smoking behavior may be transmitted through mental health and social support. To further investigate these mediating channels, we extended the baseline specification and implemented a two-step mediation test following Jiang’s approach (40), with the mediation model formulated as follows:


$${\mathrm{Med}}_{i}=a_{0}+a_{1}X_{i}+a_{2}C_{i}+\varepsilon_{i}$$ (2)

Where $\mathrm{Me}d_{i}$ denotes the mediating variable. The definitions of the remaining variables are identical to those in Equation 1.

#### 2.3.3 Moderation effect model

Building on the preceding theoretical analysis, we tested whether individual income moderates the effect of physical exercise on smoking behavior. We augmented the baseline regression by adding an interaction term between physical exercise and individual income. The moderating specification is formulated as follows:


$$Y_{i}=a_{0}+a_{1}X_{i}+a_{2}R_{i}+a_{3}X_{i}\times R_{i}+a_{4}C_{i}+\varepsilon_{i}$$ (3)

Where $R_{i}$ denotes the moderating variable, individual income; $a_{3}$ is the coefficient on the interaction term, and the remaining variables are defined as in Equation 1.

## 3 Results and analysis

### 3.1 Baseline regression result

As shown in Table 3, column (1) reports OLS estimates without covariates, while columns (2)–(4) sequentially add individual-, household-, and region-level controls. In column (1), the coefficient on physical exercise is 0.0246 (*p* < 0.05), indicating a positive association with a higher non-smoking score. After incorporating the control sets, the estimated coefficients are 0.0306, 0.0296, and 0.0276, respectively, each significant at the 1% level. The stepwise increase in *R*^2^ indicates that model fit improves as controls are introduced. Overall, the results are consistent with the theoretical expectation that participation in physical exercise is positively associated with reduced smoking behavior among Chinese adults, thereby supporting H1.

**Table 3:** Baseline regression results.

Variables	Smoking behavior			
	(1)	(2)	(3)	(4)
Physical exercise	0.0246^**^ (0.0215)	0.0306^***^ (0.0006)	0.0296^***^ (0.0010)	0.0276^***^ (0.0021)
Gender		−0.9783^***^ (0.0000)	−0.9782^***^ (0.0000)	−0.9746^***^ (0.0000)
Age		0.0049 (0.9131)	0.0157 (0.7287)	0.0167 (0.7122)
Age^2		−0.0007 (0.6785)	−0.0011 (0.5338)	−0.0014 (0.4248)
Household registration		0.0406 (0.2121)	0.0465 (0.1567)	0.0665^**^ (0.0446)
Religious belief		−0.1027^**^ (0.0205)	−0.1002^**^ (0.0239)	−0.0904^**^ (0.0415)
Educational attainment		0.0799^***^ (0.0000)	0.0714^***^ (0.0001)	0.0691^***^ (0.0001)
Marriage status			−0.0375 (0.2460)	−0.0398 (0.2185)
Household income rating			0.0291 (0.1508)	0.0294 (0.1475)
Household risk investment status			−0.0338 (0.5006)	0.0012 (0.9811)
GDP per capita				0.0937 (0.3885)
Regional per capita disposable income				0.0418 (0.7530)
Constant	2.3919^***^ (0.0000)	2.7324^***^ (0.0000)	2.7182^***^ (0.0000)	1.1858^**^ (0.0243)
*N*	2,373	2,373	2,373	2,373
Adj. *R*^2^	0.0018	0.3414	0.3417	0.3439

*p*-values in parentheses; ^*^p < 0.1; ^**^p < 0.05; ^***^p < 0.01.

### 3.2 Robustness checks

To ensure the reliability and robustness of the baseline model estimates, we implemented three validation strategies: changing the estimation method for the independent variable, employing alternative regression specifications, and expanding the set of control variables.

First, we modified the operationalization of the independent variable. Physical exercise was recorded as a dichotomous indicator (0 = “never,” 1 = otherwise) and estimated using OLS [see Table 4, column (1)]. Under this alternative coding, the coefficient on physical exercise is positive and statistically significant at the 1% level.

**Table 4:** Robustness checks.

Variables	Smoking behavior				
	(1)	(2)	(3)	(4)	(5)
	Ols model	Ordered logit model	Ordered probit model	Tobit model	Additional controls
Physical exercise	0.0895^***^ (0.0050)	0.0888^***^ (0.0047)	0.0548^***^ (0.0033)	0.0276^***^ (0.0020)	0.0331^**^ (0.0255)
Gender	−0.9735^***^ (0.0000)	−3.2356^***^ (0.0000)	−1.8243^***^ (0.0000)	−0.9746^***^ (0.0000)	−0.9502^***^ (0.0000)
Age	0.0143 (0.7514)	0.0572 (0.7316)	0.0518 (0.5927)	0.0167 (0.7115)	0.0077 (0.9195)
Age^2	−0.0010 (0.5577)	−0.0047 (0.4603)	−0.0037 (0.3192)	−0.0014 (0.4236)	0.0004 (0.8931)
Household registration	0.0648^**^ (0.0500)	0.2260^*^ (0.0543)	0.1369^**^ (0.0475)	0.0665^**^ (0.0441)	0.1647^***^ (0.0032)
Religious belief	−0.0910^**^ (0.0399)	−0.3509^*^ (0.0639)	−0.2328^**^ (0.0331)	−0.0904^**^ (0.0410)	−0.0957 (0.2450)
Educational attainment	0.0683^***^ (0.0001)	0.2703^***^ (0.0000)	0.1560^***^ (0.0000)	0.0691^***^ (0.0001)	0.0865^***^ (0.0037)
Marriage status	−0.0396 (0.2210)	−0.0980 (0.4087)	−0.0560 (0.4172)	−0.0398 (0.2174)	−0.0424 (0.4618)
Household income rating	0.0288 (0.1561)	0.1011 (0.1602)	0.0514 (0.2194)	0.0294 (0.1465)	0.0036 (0.9148)
Household risk investment status	0.0029 (0.9558)	0.0227 (0.9006)	0.0229 (0.8307)	0.0012 (0.9811)	−0.0744 (0.3633)
GDP per capita	0.0961 (0.3767)	0.3111 (0.4257)	0.1610 (0.4839)	0.0937 (0.3873)	0.1796 (0.3148)
Regional per capita disposable income	0.0386 (0.7720)	0.1949 (0.6891)	0.1479 (0.6100)	0.0418 (0.7524)	0.0151 (0.9461)
Ethnicity	-	-	-	-	0.0174 (0.8735)
BMI	-	-	-	-	−0.0014 (0.8500)
Health insurance participation	-	-	-	-	0.2922^***^ (0.0039)
Constant	1.1830^**^ (0.0247)	-	-	1.1858^**^ (0.0240)	0.2208 (0.8090)
*N*	2,373	2,373	2,373	2,373	1,113
Pseudo *R*^2^/Adj. *R*^2^	0.3435	0.2495	0.2448	0.1714	0.2570

*p*-values in parentheses; ^**^p < 0.05; ^***^p < 0.01.

Second, we replaced the regression model. Using ordered logit, ordered probit, and tobit models, we re-examined the effect of physical exercise on smoking behavior. As reported in Table 4, columns (2)–(4), the coefficient on physical exercise is positive and statistically significant at the 1% level across all specifications.

Third, we expanded the set of control variables. Specifically, we included ethnicity, BMI, and health insurance participation as covariates and re-estimated the models; results are reported in column (5) of Table 4. The coefficient on physical exercise remains positive and statistically significant at the 5% level.

Taken together, these robustness checks further corroborate the reliability and robustness of our findings.

### 3.3 Heterogeneity tests

Given China’s vast geographic diversity and a population of roughly 1.4 billion, it is important to assess whether this relationship varies across subpopulations. Accordingly, we conducted heterogeneity analyses by age and gender.

As shown in columns (1) and (2) of Table 5, we restricted the heterogeneity analysis to respondents with complete information on age, smoking behavior, and covariates, and divided the sample into three age groups: 18–34, 35–64, and ≥65 years. This grouping follows demographic and public health practice in China, where 18–34-year-olds are typically classified as youth or early adults, 35–64-year-olds as prime working-age/middle-aged adults, and those aged ≥65 years as older adults who have generally reached or passed the statutory retirement age. The cut-off points also reflect clear differences in smoking prevalence, health-risk profiles, and role responsibilities across the life course, while ensuring sufficient sample sizes and stable estimates for each subsample. For these three age groups, the estimated association between physical exercise and smoking behavior is statistically significant at the 1% level for middle-aged adults and at the 10% level for youth, but not significant among older adults. This pattern likely reflects that middle-aged adults in China exhibit higher smoking prevalence and greater daily cigarette consumption; thus, a comparable increase in exercise yields a larger absolute reduction in cigarettes smoked. Furthermore, under the combined pressures of routine health checkups, chronic-disease warnings, and smoke-free workplace policies, middle-aged individuals are more inclined to treat exercise together with smoking control/cessation as part of adopting a healthier lifestyle, thereby amplifying the marginal effect of exercise on reducing smoking. Among youth, smoking behavior is strongly shaped by social networks and peer influence. Physical exercise can suppress youth smoking to some extent by improving affect and by fostering “non-smoking” social circles. However, evidence shows that friends’ smoking substantially affects one’s own smoking (41), so pro-smoking peer norms may attenuate the net effect; accordingly, the effect for youth is statistically significant but less robust than for middle-aged adults. Among older adults, smoking habits are often deeply entrenched (sometimes spanning decades), physiological dependence is stronger (e.g., greater nicotine dependence), and loneliness reduces willingness to change; as a result, the inhibitory effect of exercise is more easily offset by other factors. Furthermore, age-related slowing of metabolism and a higher burden of chronic diseases mean that the benefits of exercise may be counteracted by health problems (e.g., arthritis), limiting its ability to reduce smoking urges effectively.

**Table 5:** Heterogeneity tests.

Variables	Smoking behavior				
	(1)	(2)	(3)	(4)	(5)
	Youth	Middle-aged adults	Older adults	Male	Female
Physical exercise	0.0436^*^ (0.0711)	0.0338^***^ (0.0072)	0.0079 (0.5907)	0.0359^**^ (0.0342)	0.0167^**^ (0.0407)
Constant	1.9714^*^ (0.0890)	0.2327 (0.7556)	1.7047^*^ (0.0681)	−0.9016 (0.3822)	1.9698^***^ (0.0000)
Control variables	Yes				
*N*	464	1,227	682	1,091	1,282
Adj. *R*^2^	0.3003	0.3465	0.3847	0.0457	0.0152

*p*-values in parentheses; ^*^p < 0.1; ^**^p < 0.05; ^***^p < 0.01.

Columns (4) and (5) of Table 5 show that the effect of physical exercise on smoking behavior is statistically significant for both men and women, with a larger coefficient for men than for women (0.0359 > 0.0167). One explanation is that China’s male-dominated social norm of “offering/receiving cigarettes” fosters smoking in social interactions, whereas participation in exercise provides men with alternative social networks and smoke-free contexts that weaken such socially cued smoking (42). Among women, some are more likely to view smoking as a weight-control strategy and are more sensitive to post-cessation weight gain, which increases relapse risk; these factors can attenuate the net effect of exercise on reducing or quitting smoking and may even lead to a pattern in which exercise and smoking occur concurrently (43).

### 3.4 Endogeneity tests

To mitigate potential confounding and sample selection bias, we re-examined the effect of physical exercise on smoking using PSM. We matched the treatment group (exercise participants) to the control group (non-participants) with three algorithms: nearest neighbor matching (*k* = 1), radius matching (caliper = 0.01), and kernel matching. As reported in Table 6, post-matching diagnostics indicate good model fit with no systematic differences between groups, and the balancing assumption is satisfied. The estimated average treatment effects are 0.1967 (significant at the 1% level), 0.1337 (5% level), and 0.1399 (5% level) for nearest neighbor, radius, and kernel matching, respectively. These results suggest that, even after addressing selection bias, participation in physical exercise remains positively and significantly associated with reduced smoking behavior, thereby further corroborating H1.

**Table 6:** PSM results.

Matching methods	Exercise participants	Non-participants	ATT	S.E.	*t*
Nearest neighbor	2.4777	2.2811	0.1967^***^	0.0671	2.93
Radius matching	2.4777	2.3440	0.1337^**^	0.0559	2.39
Kernel matching	2.4777	2.3378	0.1399^**^	0.0564	2.48

The independent variable “physical exercise” is coded as a binary indicator. Nearest-neighbor matching uses 1:1 matching with replacement, and the radius (caliper) for radius matching is set to 0.01. Owing to space limitations, balance diagnostics for the matched samples are not reported; ^**^p < 0.05; ^***^p < 0.01.

### 3.5 Mediation effect tests

The preceding analyses corroborate the finding that participation in physical exercise is positively associated with reduced smoking behavior, and the findings remain robust across multiple checks. Building on the preceding theoretical analysis, physical exercise may be indirectly associated with smoking behavior through mental health and social support. Following Jiang (40), we adopted a two-step mediation framework to examine these mechanisms, as specified in Equation 2. When the causal relation between the mediator and the outcome is theoretically specified, mediation testing needs to focus only on the effect of the independent variable on the mediator. The mediation results are reported in Table 7. As indicated by column (1) of Table 7, together with the baseline regressions, physical exercise is positively associated with reduced smoking behavior among residents; however, the specific pathways of influence require further verification.

**Table 7:** Tests of mediation effects.

Variables	(1)	(2)	(3)
	Smoking behavior	Mental health	Social support
Physical exercise	0.0276^***^ (0.0021)	0.0358^**^ (0.0125)	0.1012^***^ (0.0000)
Gender	−0.9746^***^ (0.0000)	0.2153^***^ (0.0000)	0.0679^*^ (0.0863)
Age	0.0167 (0.7122)	−0.1277^*^ (0.0636)	−0.0576 (0.3801)
Age^2	−0.0014 (0.4248)	−0.0007 (0.8027)	−0.0098^***^ (0.0002)
Household registration	0.0665^**^ (0.0446)	−0.0771 (0.1223)	−0.1116^**^ (0.0190)
Religious belief	−0.0904^**^ (0.0415)	0.1201 (0.1294)	0.1069 (0.1458)
Educational attainment	0.0691^***^ (0.0001)	0.0248 (0.3534)	0.0876^***^ (0.0003)
Marriage status	−0.0398 (0.2185)	0.1628^***^ (0.0009)	−0.2820^***^ (0.0000)
Household income rating	0.0294 (0.1475)	0.2713^***^ (0.0000)	0.1384^***^ (0.0000)
Household risk investment status	0.0012 (0.9811)	0.3015^***^ (0.0000)	−0.0491 (0.4544)
GDP per capita	0.0937 (0.3885)	−0.4529^***^ (0.0044)	0.2782^*^ (0.0606)
Regional per capita disposable income	0.0418 (0.7530)	0.7126^***^ (0.0003)	−0.3200^*^ (0.0827)
Constant	1.1858^**^ (0.0243)	0.4916 (0.5474)	2.3432^***^ (0.0019)
*N*	2,373		
Adj. *R*^2^	0.3439	0.0839	0.1665

*p*-values in parentheses; ^**^p < 0.05; ^***^p < 0.01.

As shown in column (2) of Table 7, the coefficient on physical exercise is 0.0358 and statistically significant at the 5% level, indicating a positive association between physical exercise and mental health. According to the broaden-and-build theory of positive emotions, the positive affect generated by exercise helps individuals cultivate a more adaptive mindset, thereby alleviating depressive and dysphoric states (44). Exercise is widely used as an intervention for depression; by reducing stress, improving mood, and strengthening physical health, it enhances psychological resilience. A large body of evidence has shown that physical activity lowers the risk of depression and anxiety (21, 45), promotes overall well-being (46), and helps patients rebuild positive life attitudes and social functioning. In turn, better mental health helps suppress smoking behavior, increases the likelihood of successful cessation, and reduces the risk of nicotine dependence (47). Smoking prevalence is up to three times higher among people with serious mental illnesses (e.g., depression, anxiety disorders, bipolar disorder), who often smoke to cope with stress or negative affect (48). Even after a successful quit attempt, individuals with depression may relapse if underlying mood problems persist, suggesting that cessation interventions should integrate psychological treatment (49). Consistent with the theory of planned behavior, evidence indicates that greater positive affect and higher autonomous motivation are associated with stronger intentions to quit and better follow-through (50). Accordingly, physical exercise is associated with lower smoking through improvements in mental health, thereby supporting H2.

As shown in column (3) of Table 7, the coefficient on physical exercise is 0.1012 and statistically significant at the 1% level, indicating a positive association between physical exercise and the level of social support among. This is plausibly because many forms of exercise occur in team or community settings, through which participants build social networks, gain platforms for communication (51), broaden their social ties (52), and foster interpersonal and generalized trust (28). Exercise may also mitigate negative social dispositions and expand access to supportive resources that facilitate personal goals (53). Consistent with prior evidence, social support plays a critical role in suppressing smoking and improving cessation success (54). One line of research shows that smokers’ network structure predicts quit outcomes: higher proportions of non-smokers and more supportive ties are associated with higher abstinence rates (55). Individuals with supportive partners also achieve better cessation outcomes; for instance, among Latino smokers, quit rates were 30% with partner support versus 14.3% without (56). Conversely, negative social interference (e.g., smoking prompts, lack of understanding) is linked to cessation failure (57). Accordingly, physical exercise is associated with lower smoking through enhanced social support, thereby supporting H3.

### 3.6 Moderating effect tests

Consistent with our theoretical analysis, the impact of physical exercise differs across residents with varying economic backgrounds. Income level may positively moderate the relationship between physical exercise and reduced smoking; that is, residents with higher incomes exhibit a more pronounced positive association between exercise participation and lower smoking. We proxied personal income with annual income (measured in units of 10,000 yuan) and constructed an interaction term between physical exercise and personal income to test moderation, as specified in Equation 3. As reported in Table 8, the coefficient on the interaction term (Physical exercise × Individual income) is positive and statistically significant at the 10% level, and the main effect of exercise remains positive and significant. This indicates that personal income indeed exerts a statistically significant positive moderating effect on the association between physical exercise and reduced smoking; specifically, as residents’ income rises, the positive association between exercise participation and lower smoking becomes stronger, thereby supporting H4.

**Table 8:** Tests of moderation effects.

Variables	Smoking behavior
Physical exercise	0.0226^**^ (0.0145)
Individual income	−0.0039 (0.1108)
Physical exercise×Individual income	0.0016^*^ (0.0740)
Constant	1.1487^**^ (0.0290)
Control variables	Yes
*N*	2,373
Adj. *R*^2^	0.3442

*p*-values in parentheses; ^*^p < 0.1; ^**^p < 0.05.

## 4 Discussion

Using baseline regressions, robustness checks, heterogeneity analyses, endogeneity tests, and mediation and moderation analyses, this study corroborates the effect of physical exercise on residents’ smoking behavior. We now discuss the results point by point.

This study observed an association between participation in physical exercise (PE) and a lower likelihood of current smoking among Chinese adults, consistent with prior research (11–13).

Potential pathways should be viewed as plausible rather than causal, given our cross-sectional design. PE correlates with better mood and fewer anxiety/depressive symptoms, which in turn relate to lower smoking propensity (10–13). Among smokers, higher self-efficacy, a robust predictor of cessation, is frequently observed alongside regular PE, suggesting a self-regulatory pathway (58). Experimental evidence indicates that acute PE can reduce cigarette craving and withdrawal for up to ~50 min post-exercise, with large standardized mean differences for desire-to-smoke and strength-of-desire in an individual-participant-data meta-analysis (59) and consistent findings across systematic reviews (60). In everyday contexts, a time-substitution explanation is also feasible; time allocated to PE may reduce exposure to smoking cues and opportunities to smoke (60). Simultaneously, the evidence base is not uniform. High-quality syntheses of randomized trials conclude that adding exercise to standard cessation support does not clearly improve long-term abstinence compared with support alone, with low-to-very-low certainty estimates and adherence challenges (14). Individual trials similarly report no significant differences in prolonged abstinence when exercise counseling is added to usual care, despite short-term symptom relief (61). Meta-analytic work suggests that any cessation benefits are inconsistent and may depend on exercise intensity and adherence (15); combining PE with pharmacotherapy (e.g., nicotine replacement therapy) appears to improve short-term craving/withdrawal but still shows uncertain long-term advantages (62). Heterogeneity by sex, baseline nicotine dependence, and intervention intensity has been noted across trials and reviews (14, 62).

Furthermore, this study modestly extended the explanatory framework by positing two mediating mechanisms—mental health and social support—and one moderating mechanism—individual income—and provided empirical evidence consistent with these pathways.

Physical exercise is associated with better mental health and lower smoking propensity, a pattern consistent with prior evidence (8, 22). Acute bouts of exercise reduce negative affect and craving for up to 50 min, with large standardized effects in individual-participant meta-analysis and convergent systematic reviews (60). Neurobiological studies suggest transient modulation of emotion-regulation circuits (e.g., prefrontal cortex, amygdala) and endogenous opioids/dopamine (63). With sustained participation, exercise is linked to fewer anxiety/depressive symptoms (64). These observations support a plausible emotion-regulation pathway by which better mood and reduced cue-reactivity co-occur with lower smoking.

The association between physical exercise and lower smoking appeared to be partly accounted for by higher levels of social support, a conclusion consistent with most prior studies (26, 30). Physical activity, especially group-based exercise, typically involves social interaction and relationship building; the resulting sense of connectedness provides encouragement and support when individuals face the challenges of quitting. On the one hand, smokers who participate in organized sport or fitness programs develop stronger social ties and a sense of mutual aid, which helps relieve stress and enhances cessation confidence and self-efficacy (65). On the other hand, immersion in smoke-free, physically active social milieus increases susceptibility to normative influence. In contexts such as community fitness and team sports, peers’ health-oriented attitudes and behaviors shape individual conduct through social-norm mechanisms, fostering positive imitation and reinforcement and reducing the social acceptability of smoking (9).

Moreover, mental health and social support are unlikely to operate in isolation; rather, there is a plausible interaction mechanism between the two. The stress-buffering perspective suggests that supportive social relationships can mitigate psychological distress and protect against depression and anxiety (66, 67), thereby indirectly lowering smoking motivation that is driven by negative affect. Empirical research also indicates that individuals with higher perceived social support tend to exhibit better psychological well-being (68), whereas poor mental health may erode perceived support, reduce help-seeking, and weaken the ability to mobilize existing social networks (69). Within exercise contexts, group-based activities may simultaneously enhance social support and improve mood, creating a mutually reinforcing loop in which stronger support promotes better mental health, and better mental health facilitates the formation and maintenance of supportive, health-oriented relationships. In this study, we treated mental health and social support as parallel mediators for empirical tractability; however, the above evidence implies that sequential pathways are also theoretically plausible. Future longitudinal or experimental studies should explicitly test these chain-mediating and interaction mechanisms to clarify how mental health and social support jointly shape the impact of physical exercise on smoking behavior.

The smoking-suppressing effect of physical exercise appeared stronger at higher income levels, a conclusion broadly consistent with prior research (32, 34). As a substitute behavior, exercise helps buffer affective fluctuations and physiological urges during cessation. Compared with lower-income groups, higher-income individuals are more likely to possess the resources (e.g., time, conducive environments) to initiate and sustain regular exercise; they also have greater autonomy and cognitive resources to adopt and adhere to health interventions. Consequently, the exercise-induced cessation-promoting effect is more pronounced among higher-income residents.

Moreover, we observed heterogeneity by gender and age. First, the effect of physical exercise on reducing smoking behavior is more pronounced among men than among women. A plausible explanation is that men tend to emphasize physiological reinforcement and substitutive gratification during exercise (e.g., relief of withdrawal symptoms) (70), whereas women’s exercise behavior is more sensitive to weight-management and affective considerations (71, 72), which may attenuate the net impact of exercise on cessation. Second, the effect is stronger for middle-aged adults than for other age groups, a pattern broadly consistent with prior findings (11). Conversely, among older adults, the estimated effect is small and statistically insignificant. This may reflect both social–cultural and physiological characteristics of the older adults. Many older Chinese adults began smoking in early adulthood, have accumulated decades of smoking history, and often remain embedded in social networks and family environments where smoking is culturally tolerated or even used to maintain social ties (e.g., gifting and sharing cigarettes). Under such conditions, occasional or moderate exercise may be insufficient to counteract long-standing habits and strong nicotine dependence. Furthermore, age-related declines in physical function, multimorbidity, and pain or mobility limitations can restrict the intensity and duration of exercise, thereby reducing its potential to relieve withdrawal symptoms, improve affect, or restructure daily routines around non-smoking activities. Some older adults may also experience fatigue or discomfort during exercise, reducing adherence and dampening any auxiliary cessation benefits. These social, cultural, and physiological factors help explain why the inhibitory effect of physical exercise on smoking behavior appears weaker and statistically insignificant among the older adults in our sample.

### 4.1 Limitations

Despite its theoretical and practical implications, this study has several limitations.

First, the data used in this study are cross-sectional. Although we applied multiple strategies to probe the relationship between physical exercise and smoking, as well as potential mediating pathways, the inherent limitations of cross-sectional designs restrict causal inference and our ability to capture the dynamic, long-term relationship between exercise and smoking behavior. Future research should adopt longitudinal cohort designs and experimental or quasi-experimental approaches to track behavioral changes over time and to evaluate the long-term effects of interventions and policy measures.

Second, measurement of physical exercise was constrained by the CGSS 2021 questionnaire. Physical exercise was measured by a single self-reported item on past-year participation frequency. Such self-reports are vulnerable to recall error and social desirability bias, which may misclassify true activity levels and attenuate observed associations. Future research will need to incorporate fieldwork and dedicated surveys to construct a multidimensional measure that covers duration, intensity, years of participation, activity type, and mode of participation. Objective indicators (e.g., accelerometers or wearable-device logs) and short activity diaries could further reduce misclassification.

Third, although we controlled for a range of sociodemographic and health-related covariates, the analysis remains vulnerable to the influence of unmeasured variables. For example, information on smoking history (e.g., age of smoking initiation, cumulative smoking duration, prior quit attempts, and degree of nicotine dependence) and on the family smoking atmosphere (e.g., parental or spousal smoking, home smoking rules) was limited or unavailable in CGSS 2021. These factors may shape both participation in physical exercise and smoking behavior, potentially biasing the estimated associations. Future studies should collect richer behavioral and familial data and, where feasible, apply more stringent strategies to address confounding.

Fourth, although CGSS is a nationally representative survey, regional cultural differences within China may still affect the observed relationships. Smoking norms, attitudes toward physical exercise, local tobacco-control regulations, enforcement intensity, and urban–rural contexts differ markedly across provinces and regions. Our models may not fully capture this contextual heterogeneity, and the estimated average effects could mask substantial regional variation. Future research could incorporate multi-level modeling, region-specific analyses, or additional contextual indicators (such as local smoking norms and policy stringency) to better account for these cultural and institutional differences.

Fifth, this study offered a preliminary account of how physical exercise might relate to lower smoking likelihood through several pathways, including mental health and social support. These mediating processes may interact in complex ways and may operate differently across population subgroups, and evidence from long-term follow-up remains limited. Future work should employ more precise experimental and longitudinal designs, use advanced measurement technologies, and integrate interdisciplinary methods to investigate these mechanisms in greater depth.

### 4.2 Recommendations

Based on the study’s findings, we propose several recommendations for practice and for future research.

First, actively promote the adjunctive integration of physical exercise with health education. In building and refining the health-education system, the potential supportive role of exercise in tobacco-control interventions should be emphasized as a complement, rather than a substitute for established cessation therapies (e.g., counseling, NRT, pharmacotherapy). Specifically, medical institutions at all levels, community health service centers, and other relevant health-care settings can disseminate and field-test exercise prescriptions within comprehensive cessation programs. Through professional guidance and structured education, smokers should be encouraged to adopt evidence-based, appropriately dosed physical-activity programs and to develop sound health beliefs, while rigorously evaluating uptake, safety, and short-term craving relief. Future research could embed randomized or quasi-experimental exercise components into existing cessation services to test whether different exercise “doses” or formats (e.g., group-based vs. individual programs, aerobic vs. mixed modalities) produce measurable reductions in smoking frequency and relapse.

Second, account for income heterogeneity and implement differentiated health interventions. Given the observed (non-causal) positive moderation by income in the exercise–smoking association, health support for low-income groups should be strengthened. Policies may include low-cost or free time slots at public sports facilities, issuance of exercise vouchers or point-based subsidies, and employer-based fitness incentives. Building on this, future research should conduct targeted intervention experiments for different income groups. For example, researchers could design and evaluate tailored exercise-based cessation packages for low-, middle-, and high-income participants, compare participation rates and cessation outcomes across income strata, and assess the cost-effectiveness of subsidy schemes or facility-access policies. Clustered community or workplace trials could further clarify which intervention models are most effective and scalable for disadvantaged groups.

## 5 Conclusion

Against the backdrop of the Healthy China initiative and the National Fitness Strategy, examining the associations and potential mechanisms linking physical exercise with residents’ smoking behavior is theoretically and practically significant. Using data from CGSS 2021, this study employed OLS regressions along with robustness, heterogeneity, and endogeneity tests to assess the associations between exercise and smoking. We further explored mediation through mental health and social support and tested the moderating role of personal income. The results showed that (1) Participation in physical exercise was associated with a lower likelihood of residents’ smoking behavior. This finding remained robust after re-operationalizing the key explanatory variable, altering model specifications, adding control variables, and addressing selection bias via PSM on observed characteristics. (2) The negative association between exercise and smoking exhibited heterogeneity by gender and age, appearing stronger among men and among middle-aged adults within subgroup analyses. (3) Mediation analyses were consistent with the possibility that exercise relates to lower smoking partly via better mental health and greater social support. (4) Moderation analyses suggested that individual income positively moderated the exercise-smoking association; higher-income individuals showed a more pronounced negative association in this sample.

## Data Availability

Publicly available datasets were analyzed in this study. This data can be found here: http://cgss.ruc.edu.cn/.
